# Health-Related Quality of Life Assessment for Liver Cirrhosis Patients at a Tertiary Care Hospital in Karachi, Pakistan

**DOI:** 10.7759/cureus.53766

**Published:** 2024-02-07

**Authors:** Darayus P Gazder, Syed Shahrukh Parvez, Noshirwan P Gazder, Jamil Muqtadir

**Affiliations:** 1 Gastroenterology and Hepatology, Ziauddin University, Karachi, PAK; 2 Pulmonology, Ziauddin University, Karachi, PAK; 3 Infectious Diseases, Ziauddin University, Karachi, PAK

**Keywords:** pakistan, quality of life, physical functioning, health-related quality of life, cirrhosis

## Abstract

Background

The global health challenge of cirrhosis affects millions of individuals. Still, there is a notable lack of research on the health-related quality of life (HRQOL) of cirrhosis patients, especially in specific regions. This study aims to assess the impact of cirrhosis on HRQOL, including factors influencing it in a tertiary care setting in Pakistan.

Methodology

A cross-sectional study was conducted on a cohort of 115 patients diagnosed with cirrhosis, confirmed on imaging. HRQOL was measured using the short-form 36 (SF-36) survey. Furthermore, socioeconomic factors and self-rated health perceptions regarding HRQOL were recorded. Factors Influencing HRQOL domains in liver cirrhosis patients were also analyzed.

Results

Among 115 liver cirrhosis patients, 63.5% (N=73) were aged 40-60 years, and 73.9% (N=85) lived primarily in urban areas. HRQOL assessments highlighted moderate disability in physical functioning (62.6 ± 26.5) and notable impacts on mental health, emotional well-being, and bodily pain. Statistical analysis demonstrated the complexity of factors influencing HRQOL. Physical functioning was significantly associated with a higher Child-Turcotte-Pugh class, diabetes mellitus, hypertension, smoking status, and family monthly income (p-value < 0.05). While the duration of liver cirrhosis showed a significant association with bodily pain (p < 0.05). Additionally, residence status with social well-being (p-value = 0.01), and family monthly income with vitality (p-value < 0.05).

Conclusion

Our study in Pakistan highlights significant impacts on the HRQOL of liver cirrhosis patients, affecting physical function, mental health, emotional well-being, and bodily pain. Factors influencing HRQOL include liver disease severity, comorbidities, and socio-economic status. Recommendations include educational programs and specialized nursing care to address diverse challenges. The findings stress the importance of a personalized approach to patient care, urging urgent, targeted efforts to improve the health-related standard of life for cirrhotic patients.

## Introduction

Cirrhosis is a persistent and advancing liver condition that cannot be reversed. It is accompanied by problems such as hepatic encephalopathy and upper gastrointestinal bleeding, which result in recurrent relapses and the need for hospitalization [1]. Over 500 million patients have received a diagnosis of chronic liver disease resulting from a viral etiology [2]. Furthermore, there has been a significant rise in the prevalence of chronic liver diseases attributed to non-viral factors, such as non-alcoholic and alcoholic fatty liver disease, cryptogenic hepatitis, and autoimmune hepatitis [3]. Liver cirrhosis is a primary contributor to elevated rates of death and illness in individuals with chronic liver conditions. In 2010, the mortality rate from liver cirrhosis surpassed one million individuals, a significant increase from the 0.6 million deaths recorded in 1980 [4]. Liver cirrhosis carries a substantial societal and familial burden and significantly affects the physical and emotional well-being, as well as the overall standard of life, of individuals affected by the condition [5].

Medical strategies for cirrhosis, such as the detection of hepatocellular carcinoma and monitoring of esophageal varices, primarily aim to minimize the risk associated with the condition [6]. While these clinical-focused tactics are undoubtedly significant, they overlook patient factors such as the health-related standard of life [7]. In addition to the seriousness and implications of liver cirrhosis, certain symptoms specific to the disease, such as itching, muscle cramps, sleep problems, sexual dysfunction, exhaustion, and gastrointestinal symptoms, are significant in assessing the quality of life in this group of patients [8]. The prognosis for individuals with cirrhosis can be affected by several factors, including Child-Turcotte-Pugh and Model for End-Stage Liver Disease scores, non-alcoholic etiology, and the patient's body mass index [9,10].

Patients with cirrhosis have distinct physical and cognitive symptoms that interfere with their way of life and that differentiate cirrhosis from other illnesses. Health-related aspects of life are particularly important for these patients, as the absence of medication greatly increases their chances of survival, barring liver transplantation [11]. The 36-item short-form questionnaire is a non-disease (generic) specific questionnaire that provides a composite scale of mental and physical health for patients with chronic afflictions such as gastroesophageal reflux disease, chronic liver disease, or inflammatory bowel disease [12]. The aspects of the short-form 36 (SF-36) survey include general health, physical and social functioning, bodily pain, role-physical, mental health, role-emotional, and vitality [13]. 

Cirrhosis is the primary cause of chronic liver disease in Pakistan [14], which is one of the 10 countries with the highest prevalence of liver diseases, including hepatitis, that can lead to liver fibrosis. A reduction in the general quality of life in liver cirrhosis patients correlates to poor treatment outcomes, poorer compliance to therapy, rising comorbidities, and poor prognosis of liver cirrhosis [15]. There has been a lack of extensive research conducted on evaluating the standard of life in liver cirrhosis patients in Pakistan. The scarcity of medical facilities and human resources in the healthcare sector poses a significant challenge in delivering optimal healthcare services to the public. In the existence of such entities, the healthcare is unable to provide “required” facilities and in return impacts the health state of the patients. It is crucial to understand the underlying processes that contribute to reducing late liver disease. This knowledge can help identify potential targets for future therapies that meet clinical requirements and patient demands. Rather than just treating specific symptoms, an in-depth review of various treatments for advanced liver disease should be conducted to assess their long-term health benefits. This approach can help find lasting solutions for liver disease management.

## Materials and methods

Subjects 

The research was conducted over six months, from May 26, 2023 to November 26, 2023, at the outpatient department of gastroenterology at a private institute, Ziauddin Hospital, Karachi, employing a cross-sectional design. The selection of 115 patients was made for the study's sample size by utilizing an open public calculator and employing a non-probability consecutive sampling strategy. Patients who met the inclusion criteria were enrolled in the study after obtaining ethical review committee approval from the Dr. Ziauddin University Hospital Ethical Review Committee (7500953MMED).

Patient selection

The inclusion criteria for the study comprised individuals aged between 40 and 80 years of any gender including liver cirrhosis, based on abdominal ultrasound findings. Abdominal ultrasound scans had to meet at least three of the following criteria on ultrasound: liver size reduction (longitudinal diameter of the right and left lobes less than 90 mm and 70 mm, respectively), liver nodularity on the surface, increased coarseness in liver echo texture, ascites (more than 100 mL), and portal hypertension (enlarged portal vein size more than 13 mm). On the other hand, the exclusion criteria encompassed individuals who did not give consent, had a record of hepatocellular carcinoma, depression, mania, cognitive impairment, and different chronic illnesses, including hypothyroidism, hyperthyroidism, congestive heart failure, asthma, chronic renal failure, chronic obstructive pulmonary disease, and stroke.

Data collection procedure

The recruitment of participants who met the specified criteria was conducted in the Gastroenterology Outpatient department, after obtaining the necessary approval from the institutional ethical review committee. Informed written consent was obtained from the participants, and their health status was evaluated using the SF-36 questionnaire. It comprises eight subscales including perceived mental health, physical functioning, general health perceptions, role restrictions due to physical and emotional difficulties, social functioning, vitality, and bodily discomfort. The study gathered both quantitative variables like age, SF-36 domain scores, and duration of liver cirrhosis, and qualitative variables like gender, residence status, Child-Turcotte-Pugh class, diabetes mellitus type II, hypertension, smoking, family monthly income, occupational status, educational status for analysis.

Data analysis procedure

The research analyzed demographic data, which included age, SF-36 domain scores, and duration of liver cirrhosis, using Statistical Package for the Social Sciences (SPSS) (version 26; IBM SPSS Statistics for Windows, Armonk, NY). Descriptive statistics were applied to report the data. Mean and standard deviations were used for quantitative variables. Categorical variables, such as gender, residence status, Child-Turcotte-Pugh score, diabetes mellitus type II, hypertension, smoking, family monthly income, employment status, and educational status, were presented as frequencies and percentages. To account for effect modifiers, the study categorized participants based on age, gender, residence status, Child-Turcotte-Pugh scores, diabetes mellitus type II, hypertension, smoking, family monthly income, occupational status, educational status, and duration of liver cirrhosis. This was done to assess how these factors influenced the outcome. A post-stratification independent t-test/ANOVA was performed, and statistical significance was determined at a p-value of less than 0.05.

## Results

Socio-demographic profile

The study examined 115 patients suffering from liver disease, with an average age of 57.14 ± 6.49 years. The patients, on average, had been suffering from liver cirrhosis for approximately 4.72 ± 2.24 years. Table 1 presents a comprehensive overview of the socio-demographic characteristics of the patients enrolled in the study. In terms of age distribution, 63.5% of patients are aged 40-60 years (N=73), and 36.5% (N=42) are aged 61-80 years. Gender distribution is nearly equal, with most patients 73.9% (N=85) residing in urban areas, highlighting the urban-centric nature of the study population. Duration of liver cirrhosis shows a split, with 54.8% (N=63) having a duration of two years or less. Childs Pugh classification demonstrates diverse representation, with 36.5% (N=42), 20% (N=23), and 43.5% (N=50) falling into classes A, B, and C, respectively. Diabetes mellitus type II and hypertension are prevalent in 51.3% (N=59) and 50.4% (N=58) of patients, respectively. Smoking status indicates that 22.6% (N=26) of patients are smokers. Monthly income varies, with 36.5% (N=42) earning less than 50,000 Pakistani rupees (PKR) ($1 is equivalent to around 285.4 PKR), and 61.7% (N=71) are employed. There is significant educational diversity among the respondents, with 7.8% (N=9) illiterate, 24.3% (N=28) primary educated, 41.7% (N=48) secondary educated, and 26.1% (N=30) having higher education.

**Table 1:** Socio-demographic characteristics of the patients. CTP: Child-Turcotte-Pugh; PKR: Pakistani Rupee

Characteristic	Category	Frequency (N)	Percentage (%)
Age Group	40-60 years	73	63.5
	61-80 years	42	36.5
Gender	Male	57	49.6
	Female	58	50.4
Residence Status	Urban	85	73.9
	Rural	30	26.1
Duration of Liver Cirrhosis	≤ 2 years	63	54.8
	> 2 years	52	45.2
CTP Sscore	A	42	36.5
	B	23	20.0
	C	50	43.5
Diabetes Mellitus Type II	Yes	59	51.3
	No	56	48.7
Hypertension	Yes	58	50.4
	No	57	49.6
Smoking Status	Smoker	26	22.6
	Non-smoker	89	77.4
Family Monthly Income Status	≤ 50,000PKR	42	36.5
	> 50,000PKR	73	63.5
Occupational Status	Employed	71	61.7
	Unemployed	44	38.3
Educational Status	Illiterate	9	7.8
	Primary	28	24.3
	Secondary	48	41.7
	Higher	30	26.1

Rankings of health-related quality of life (HRQOL)

Additionally, Table 2 shows the mean scores of the HRQOL domains. Physical functioning exhibited the highest mean score at 62.60±26.5, suggesting a moderate level of disability. Mental health was closely tracked with a mean score of 57.35±15.3, revealing considerable interplays between liver cirrhosis and psychological features. Role emotional (59.90±30.5) and bodily pain (58.13±29.8) scores indicate issues in emotional well-being and discomfort, respectively. Social functioning (53.60±19) highlights mild impacts on social interactions. General health (46.01±24.1) depicts a compromised overall well-being. Vitality (46.12±38.2) and role physical (38.91±31.27) scores reflect issues in energy levels and everyday activities, respectively.

**Table 2:** The mean scores of each health-related quality of life domains with their respective standard deviation. HRQOL: Health-related quality of life; SD: standard deviation

HRQOL domains	Mean	±SD
Physical functioning	62.6	26.5
Role emotional	59.9	30.5
Bodily Pain	58.13	29.8
Mental health	57.35	15.4
Social functioning	53.6	19.0
Vitality	46.12	38.2
General-health	46.01	24.2
Role physical	38.91	31.3

Factors influencing HRQOL domains in liver cirrhosis patients

Moreover, Table 3 illustrates the significant associations between various factors and aspects that impact the overall welfare of individuals diagnosed with liver cirrhosis. It was observed that Child-Turcotte-Pugh class, diabetes mellitus, hypertension, smoking status, and monthly income showed significant associations with physical functioning. Similarly, variables such as advancing age, gender, residence status, duration of liver sickness, Child-Turcotte-Pugh class, hypertension, diabetes mellitus, tobacco use, income per month, and level of education exhibited significant relationships with other domains related to health aspects of life, including the physical role, bodily pain, general health, social functioning, role emotional, vitality, and mental health. The data indicates a wide range of factors that affect the lives of patients with liver impairment.

**Table 3:** Substantial correlations among distinct variables and diverse domains that influence the well-being of individuals diagnosed with liver cirrhosis. HRQOL: Health-Related Quality of Life; CTP score: Child-Turcotte-Pugh score

HRQOL domain	Significant associations
Physical functioning	CTP score, Diabetes Mellitus, Hypertension, Smoking Status (p-value<0.01), Family Monthly Income (p-value <0.05)
Role physical	Gender (p-value=0.01)
Bodily pain	Duration of Liver Cirrhosis (p-value<0.05)
Mental wellness	Age (p-value<0.05)
Social wellbeing	Residence Status (p-value=0.01)
Vitality	Family Monthly Income (p-value<0.05)
General-health	Age (p-value<0.05)
Role physical	Gender (p-value=0.01)

## Discussion

Cirrhosis is progressively more prevalent in both industrialized and developing societies, resulting in elevated levels of morbidity and mortality. This has brought recognition to it as a significant global public health concern. The severity and progressive nature of the disease significantly impact those affected. The presence of liver cirrhosis exacerbates treatment results and correlates to negative patient-reported outcomes. The current analysis indicated a poor quality of life among individuals with the condition. It highlighted the significant impact of cirrhosis on various aspects of health-related well-being, with physical and social functioning showing the highest scores.

Pradhan et al. conducted a study on 60 individuals with chronic liver disorders to assess their health-associated standard of life [16]. The primary factors measured in this study were physical functioning (PF: mean of 34.4 ± 26.7standard deviation), role limitation due to physical health (RLPH: 7.5±17.8), role limitation due to emotional problems (RLEP: 27.7±38.2), energy or fatigue (E/F: 38.5±21.5), emotional well-being (EWB: 57.7±22.8), social functioning (SF: 55.2±23.5), pain (44.8±30.3), and general health (GH: 38.2±17). Employment status and a higher annual family income were found to be linked to positive improvements in the standard of life. It has been observed that poor health status perceptions are common among patients with ascites, abnormal upper gastrointestinal endoscopic findings, and higher Child-Turcotte-Pugh class. A negative association was found between the model for end-stage liver disease scores and declining health-associated standards of life with increasing disease severity (p-value<0.05). While our research and other studies provide valuable insights into the health-related aspects of the life of cirrhotic patients, differences in methodology, sample sizes, and demographics may lead to discrepancies in reported results. Understanding the various factors that impact a disease is crucial in comprehending its complex nature. This knowledge opens up avenues for additional investigation and intervention [16].

Janani et al. conducted a study on 149 patients with liver cirrhosis. They compared their SF-36 survey scores with those of age and gender-matched controls [11]. The study revealed that the individual and composite domain scores of the cirrhotic patients, except for physical pain, were significantly lower (p-value < 0.0001). Patients who were below 45 years of age, particularly those with a high model of end-stage liver disease and Child-Turcotte-Pugh scores, experienced increased rates of complications and lower SF-36 ratings for physical pain (p-value < 0.005). The study found that physical components had a significant impact on the mental composite score (p-value <0.05). People under the age of 45 also had a low overall chronic liver disease questionnaire score (p-value < 0.05). Diabetes, with or without other co-morbid conditions, did not affect SF-36 or chronic liver disease questionnaire scores. However, non-diabetic co-morbid illnesses had an impact on physical domains (physical function, body discomfort, and role physical) and the physical component score of SF-36 (p-value <0.01) [11].

A cross-sectional study conducted by Gazineo et al. included 254 patients who were diagnosed with chronic liver disease [17]. The mean age of the patients was 62.84 years (Standard deviation ±11.75), and 57.9% (N=147) of them were male. There were 40.2% of patients with compensated cirrhosis and chronic hepatitis (N = 102 in both groups), and 69.3% with a disease duration exceeding five years (N = 176). Based on the model for end-stage liver disease score, 67.7% (N=172) of the patients were in Class I, 29.9% (N=76) in Class II, and 2.4% (N=6) in Class III. No patients were in Classes IV and V. The study found no significant changes in the short frequency 12 questionnaire and Nottingham health profile regions between the model for end-stage liver disease score classes, except for chronic liver disease's impact on sexual life and holidays (p-value = 0.037 and 0.032, respectively). The study also identified a prevalence rate of 26% (N = 66) for depressed symptoms. Still, there were no significant changes in Beck's Depression Inventory (BDI-II) total scores among the three categories for the model for end-stage liver disease scores [17].

The following study has a comprehensive approach to understanding the HRQOL of liver cirrhosis in a facility in Karachi, Pakistan using the 36-item short-form survey. This study is significant because it addresses a significant gap in the literature about the unique healthcare challenges and cultural factors in Pakistan, providing geographical specificity. Identifying statistically significant links between demographic and clinical characteristics and different aspects of patients' health makes this study more robust, providing a holistic view of the impact of cirrhosis. Our study has limitations due to its design, which may affect its ability to establish causation or observe longitudinal changes. The sample size could benefit from expansion to improve statistical power and generalizability. The exclusion criteria may limit the study's generalizability, considering mental health is integral to HRQOL. Being a single-center study, the findings may not universally apply to diverse healthcare settings or regions with different infrastructures. Given these collective findings, it is apparent that a full understanding of the multiple variables impacting the standard of life in patients with cirrhosis is necessary for creating targeted interventions and enhancing the overall well-being of affected persons. Further study and collaborative efforts are necessary to identify subtle aspects and optimize the effectiveness of treatment approaches in this patient population.

## Conclusions

Our study reveals significant insights regarding HRQOL based on sociodemographics and clinical factors in Pakistan. The effects on physical function, mental health, emotional well-being, and bodily pain are notable. The findings highlight the intricate interplay of factors influencing HRQOL, with associations observed between physical functioning and factors such as liver disease severity, comorbidities, and socio-economic status. Practical recommendations, including educational programs and specialized nursing care, are suggested to raise awareness and address the diverse challenges faced by these individuals. A comprehensive approach to patient care, integrating medical and non-medical interventions. The findings propose the potential for personalized treatment choices based on demographic and clinical characteristics, underscoring the urgency of tailored interventions for cirrhosis patients.
